# Redox-mediated bypass of restriction point via skipping of G1pm

**DOI:** 10.1186/1742-4682-3-26

**Published:** 2006-07-25

**Authors:** Arnold Hoffman, James J Greene, Lee M Spetner, Michael Burke

**Affiliations:** 1 Redoxia, Jerusalem, Israel; 2 Catholic University of America, Washington, DC, USA; 3 Tel Aviv Sourasky Medical Center, Tel Aviv, Israel

## Abstract

**Background:**

It is well known that cancer cells bypass the restriction point, R, and undergo uncontrolled cell proliferation.

**Hypothesis and evidence:**

We suggest here that fibrosarcoma cells enter G_1ps _directly from M, skipping G_1pm_, hence bypassing R, in response to redox modulation. Evidence is presented from the published literature that demonstrate a shortening of the cycle period of transformed fibroblasts (SV-3T3) compared to the nontransformed 3T3 fibroblasts, corresponding to the duration of G_1pm _in the 3T3 fibroblasts. Evidence is also presented that demonstrate that redox modulation can induce the CUA-4 fibroblasts to bypass R, resulting in a cycle period closely corresponding to the cycle period of fibrosarcoma cells (HT1080).

**Conclusion:**

The evidence supports our hypothesis that a low internal redox potential can cause fibrosarcoma cells to skip the G_1pm _phase of the cell cycle.

## Background

The normal cell cycle consists of four main phases; G_1_, S, G_2 _and M. G_1 _is further subdivided into two parts, G_1pm _and G_1ps _[1]. In G_1pm_, a series of mitogenic events prepares the cell to enter G_1ps _and to continue to S and M [1,2]. At the end of G_1pm_, there is a restriction point, R, which monitors the cell and checks its qualifications for entry into G_1ps_. If the accumulation of mitogenic events is inadequate, or if the cell is confluent with neighboring cells fully around its perimeter, the cell cannot pass from G_1pm _through R into G_1ps _and proliferate. Instead, the cell leaves the cell cycle and enters G_0_, the quiescent phase [1-5]. Cancer cells, on the other hand, bypass R with consequent uncontrolled proliferation [2].

Zetterberg and Larsson demonstrate that the transformed 3T3 cells, SV-3T3, behave in a similar way [3,4]. Furthermore, they demonstrate that these transformed cells do not enter G_0_. They conclude from this that tumor cells do not enter G_0 _[4].

Zetterberg and Larsson [1] have measured the duration of both G_1pm _and the complete cell cycle. Larsson and Zetterberg [3] have determined the cycle period of SV-3T3 cells. From the data in [1] and [3], we calculate that the difference between the cycle periods of the 3T3 and SV-3T3 cells is 23%; i.e. the cycle period of SV-3T3 cells is 23% shorter than that of 3T3 cells and matches the duration of G_1pm_.

We hypothesize here that the 23% decrease in cycle period of SV-3T3 is observed because these cells skip G_1pm _and enter G_1ps _directly from the exit from M. In skipping G_1pm _the SV-3T3 cells bypass R. This hypothesis is supported by the following: (1) it readily accounts for the qualitative differences between non-transformed and transformed cells as noted above; and (2) it accounts for the quantitative difference between the non-transformed and transformed cell-cycle periods.

**Figure 1:**
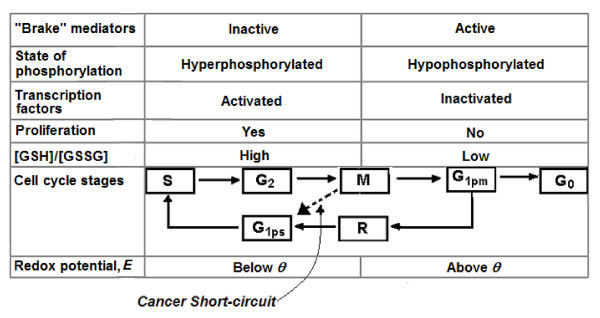
**Relationship between Rb brake and other aspects of cell cycle**. The Rb protein acts as a brake on several of the phases of the cell cycle, dependent upon its state of phosphorylation. In the hyperphosphorylated state, the Rb brake is inactive, permitting the transcription factors to become activated and cellular proliferation to proceed. During this period the ratio [GSSG]/[GSH] is low and *E *falls below *θ*. The cell passes through the restriction point R to the later stage of G_1_, termed G_1ps_, on to S, from which it passes through G_2 _to the early M phase. After mid-M, the Rb protein becomes hypophosphylated and the brake is active. The transcription factors are inactivated and cell proliferation is stopped. During this period the ratio [GSSG]/[GSH] is high and *E *rises above *θ*. The cell passes through M to the early stage of G_1_, termed G_1pm_, from which it may either return to the cell cycle via R or it passes into a resting stage, G_0_. In cancer, a portion of the cycle can be short-circuited, via the M to G_1ps _bypass. R = site of restriction point. Arrow with interrupted line represents short-circuit in cancer. *θ *= -207 ± 11 mV.

The relationship between Rb brake and other aspects of cell cycle is depicted in figure 1. The mechanism we suggest for the cancer cell skipping G_1pm _follows from our model of redox modulation of cellular proliferation [6]. Beyond the restriction point, R, the cell is committed to duplicating its DNA and proceeding to mitosis. For a cell to pass R, special proliferation-promoting proteins must be phosphorylated to promote the activation of the genes necessary for the cell to traverse R, enter G_1ps_, and proliferate. These include the retinoblastoma protein (pRb) [2,5], regulatory enzymes such casein kinase [7], and transcription factors such as jun [7] and NF-κB [8]. When the intracellular redox potential, *E*, is high, these proteins are dephosphorylated; when *E *is low they are phosphorylated [7-10].

An example of a critical phosphorylation-dependent pathway regulating passage through G_1pm _is the cyclin D-cdk4 complex. This complex phosphorylates pRb, thereby deactivating its repressor activity and allowing for transcription of S-phase genes. For this reason, the hypothesis is limited to transformed and malignant cells in which pRb is functional. According to the redox model, the dephosphorylation of pRb can occur only if the intracellular redox potential, *E*, is above a threshold value, *θ*, which we have estimated to be between -218 and 196 mV [6]. The cell normally sets *E *below *θ *when the activating proteins are to be phosphorylated, and sets *E *above *θ *when they are to be dephosphorylated [6] (see figure 1).

During normal proliferation, when the cell is in M, a phosphatase dephosphorylates pRb [5], and the transcription factors no longer become available for activating the proliferation-promoting genes. The cell then exits M and enters G_1pm _and again begins to accumulate mitogenic events necessary for the cell once more to pass R and enter G_1ps _[2].

Although multiple, often overlapping, pathways impinge on cell-cycle regulatory points, pRb is one of the key downstream elements known to play a critical regulatory role [2,5]. Since the proper functioning of the unmutated pRb is dependent on cycling between its phosphorylated and unphosphorylated states, the redox state may contribute to altering the cell cycle by affecting pRb directly or at an upstream point. According to this redox model, if E were to be below *θ *for the duration of the complete cell cycle, pRb would remain phosphorylated through the cycle thereby resulting in loss of its normal regulatory properties. Indeed, several workers have noted that the level of phosphorylated pRb is higher in cancer than in normal cells [11,12]. Our hypothesis is that some transformed and malignant cells are characterized by an *E *that is constantly below *θ*, regardless of external conditions such as confluence or growth factors. A low *E *keeps pRb from becoming dephosphorylated, preventing the passage from M into G_1pm_, but allowing their entry from M directly into G_1ps _[5], resulting in these cells skipping G_1pm_. Hutter *et al*. have reported that, in contrast to normal cells, the average *E *of fibrosarcoma cells is below the value we have estimated as *θ*, independent of the degree of confluence [13]. These cells can be thought of as internally redox-biased.

The redox model predicts that even normal cells, in which *E *is artificially maintained below *θ *for a complete cell cycle, will also skip, or short-circuit, G_1pm_. In other words, the cycle period of an artificially redox-biased normal cell will, according to the model, be shortened like the cycle period of a cancer cell. As a result of the shorter cycle period, the cell density of the redox-biased cells should be higher than that of the non-redox-biased cells. Hutter *et al*. [13] did exactly that experiment. They lowered *E *for 24 hours by adding GSH precursors. They did two experiments, adding 0.05 mM N-acetylcysteine (NAC) in the first and 0.002 mM oxothiazolidine-4-carboxylate in the second. These additions each resulted in a lowering of *E*, by 10 mV in the first and by 8 mV in the second. In both cases, the resulting *E *was below our estimated value of *θ *and in both cases the cell density increased by 26%, compared to the controls, during the 24-hour experiment. These data may be interpreted as the results of a combination of increased growth rate along with decreased sensitivity to contact inhibition. To the extent that the cells are not contact-inhibited, we can infer that they bypass R.

## Results and discussion

The doubling time, *τ*_*n*_, of various fibroblasts was measured, as was the doubling time of fibrosarcoma cells, *τ*_*c *_(see Table 1). Whereas doubling times can be dependent on the passage level in the case of non-immortal cell lines, as well as the culture medium, doubling times of normal cells were determined only at low passage and represent the lowest doubling times observed. Under these conditions it is reasonable to suppose that the observed doubling times of the fibroblasts were indeed representative of the true doubling times and did not reflect the trapping of some cells in G_0_. Table 1 shows the observed doubling time for the fibrosarcoma cells as 18.2 h and that of the CUA-4 fibroblasts as 24.1 h. Note that cancer cells do not usually enter G_0_, so all cells contribute to the proliferation and hence to the doubling time.

**Table 1:** Doubling Times of Fibroblasts and Fibrosarcoma cells^‡^

Cell Line	Cell Type	Doubling Time,*τ *(h)
CUA-4	Fibroblasts	24.1
GM08086	Fibroblasts	24.4
JHU-1	Fibroblasts	23.9
HT1080	Fibrosarcoma	18.2

^‡ ^Doubling times were determined from cell growth curves of duplicate cultures. Cell counts were made every 24 h over a period of 96 h and the standard error in the counts was <2.5% for all curves. Normal fibroblast cultures were between passage levels of 9 to 20 passages. HT1080 passage levels were in excess of 100 passages.

From Table 1, the ratio of the doubling time of the fibrosarcoma cells to that of the CUA-4 fibroblasts is

*τ*_*c*_/*τ*_*n *_= 18.2/24.1 = 0.76

Now let us calculate the doubling time of the CUA-4 fibroblasts (*τ*_*n*_) and that of he externally redox-modulated CUA-4 fibroblasts (*τ*_*e*_) from the cell-density data of Hutter *et al*. Since these latter cells do not enter G_0 _and do not exhibit contact inhibition, their observed doubling time is a measure of their cycle period.

For exponentially growing cells, it can be shown that the ratio, r, of the cell densities of the redox-biased fibroblasts compared to the non-redox-biased fibroblast controls is given by



In the experiment of Hutter *et al*., the cell density of the *E*-biased fibroblasts was 26% greater than that of the unbiased cells, so we set *r *= 1.26 in (1). The duration of their experiment was *T *= 24 h. As noted above, *τ*_*n *_for CUA-4 cells = 24.1 h. We then deduce from (1) that *τ*_*e *_= 18.1 h. The ratio of the doubling time of the *E*-biased fibroblasts to that of the unbiased fibroblasts is

*τ*_*c*_/*τ*_*n *_= 18.1/24.1 = 0.75

which compares favorably with the ratio computed above of the doubling times of fibrosarcoma cells to fibroblasts. Moreover, using the data from (1), we have computed the doubling time of the 3T3 cells to be 15.40 h and that of the cells if they had skipped G_1pm _to be 11.82 h. The ratio of these is



The close correspondence of all three of these ratios supports our hypothesis that cells in which *E *is continually below *θ *skip G_1pm_.

Although these numbers do not necessarily prove that *E*-biased normal cells skip G_1pm_, they do show that their *doubling time *is the same as that of fibrosarcoma cells. This result may be consistent with these cells bypassing R by skipping G_1pm_. Yet it is possible that the *doubling time *is shorter only because none of the cells can now enter G_0_, whereas without the *E *bias, some of the cells can enter G_0_. This possibility seems unlikely, however, because of the way the doubling times were measured. In any case, these data do demonstrate a redox-mediated bypass of R that reduces the *doubling time *of the normal cells to about 75% of its former value. Data of the sort obtained by Zetterberg & Larsson [1] for these CUA-4 cells and for the fibrosarcoma cells would help determine how closely the cycle period of normal cells corresponds to what would be the period if the normal cell would have skipped G_1pm_.

Nevertheless, there are other types of transformed cells that apparently behave differently. For example, Irani *et al*. note that 3T3 cells transformed with H-ras V12 produce superoxide constitutively, and such production is required for their uncontrolled proliferation [14]. Exposure of these cells to a reductant such as NAC inhibits their growth. In contrast, these authors report in an earlier paper that there is little if any response of Raf-transformed 3T3 cells to NAC [15]. Finkel reconciles these results [16] by suggesting that in some cells, ROS may mediate growth regulatory pathways, whereas in other cells, the data suggest that ROS play a role in apoptotic pathways. Furthermore, other data may appear to be inconsistent with our proposal of how E affects cell proliferation. For example, Szatrowski and Nathan report that ROS production is increased in cancer cells [17]. Radisky *et al*. report that experimental evidence indicates a direct link between abnormal signal transduction by oxygen species, that is redox signaling, and malignant invasive cell growth [18]. In addition, Chiarugi and Cirri point out that transduction by ROS, through reversible phosphotyrosine phosphate, is triggered by growth-factor receptors [19]. Nevertheless, none of these results show that the increase in ROS levels is sufficient to affect *E *significantly, nor do they contradict the hypothesis of bypassing G_1pm_. Indeed, attempts to measure the effect of increased physiological concentrations of ROS have been shown to be inadequate to alter cellular *E *[20] as defined here, and as seen by the molecules in the cell. The addition of NAC to a cell does not always alter the [GSSG]/[GSH] ratio (21). Martindale and Holbrook [22] conclude that at the cellular level, the response of cells to oxidants can range from proliferation, to growth arrest, to senescence, and to cell death. The particular response varies from one type of cell to the next, the agent, its dosage and duration of treatment [23].

## Conclusion

In summary, we have presented two independent sets of experimental data that are consistent with the difference between the cell-cycle period of fibroblasts and transformed SV-3T3 cells approximating the G_1pm _fraction of the cycle period of the fibroblast controls, as measured by Zetterberg and Larsson [1]. Moreover, the data demonstrate that by imposing an external E bias on the fibroblasts, they can be made to mimic the fibrosarcoma cell-cycle period and its bypassing of R. These data support our hypothesis that a low E can cause the fibrosarcoma cells to skip G_1pm_.

This hypothesis has therapeutic ramifications. Larsson *et al*. have suggested that further insight into the differences between normal and transformed cells could be useful in the search for anti-tumor agents [3]. Our hypothesis leads to the conclusion that increasing the *E *of the some tumor cells will not only prevent their skipping G_1pm_, but may result in these cells being arrested in G_1pm_, and selectively undergoing apoptosis [6]. An experiment that would partially test our theory would be to raise the *E *of proliferating fibroblasts gradually by adding a GSH-decreasing agent, such as diamide plus BCNU [23]. We predict that cell proliferation would be unaffected as *E *rises, but when *E *exceeds the value we have identified as the threshold, proliferation will come to a stop. Such a response could be exploited to provide a new treatment modality for some forms of cancer.

## Methods

The doubling times of tumor cells were measured and compared to those of proliferating normal cells. Doubling times were determined for cultures grown in Delbecco's modified minimal essential medium supplemented with 10% fetal bovine serum. This formulation was determined to provide the shortest doubling times for the cells studied. Although doubling times of these cell strains are dependent upon passage level (for fibroblasts) and the growth media used, including the brand and lot of serum, this formulation provided fairly consistent results across experiments and passage levels while retaining the relative difference between the normal cell strains and the fibrosarcoma cells. The doubling time was calculated from growth curves. For this determination, each of the cell lines was inoculated into wells of a 24-well titer plate at a density of 4 × 10^4 ^cells/well. The normal CU-4, GM08086, and JHU-1 cells were at low passage levels (population doubling 9 – 20). The CUA-4 and JHU-1 cells were derived from primary cultures of human normal foreskins at Catholic University of America, Washington, DC and the Johns Hopkins University, respectively. The normal fibroblasts GM08086 cell strain was obtained from the Coriell Institute, Camden NJ, while the HT1080 fibrosarcoma cells were obtained from American Type Culture Collection (ATCC, Manassas, VA, USA). The passage numbers for HT1080 cells at the time of the experiment was ≥100 passages. A periodic determination of doubling times for HT1080 remained fairly constant regardless of passage level. Doubling times for CUA-4 and JHU cultures were routinely measured in duplicate or triplicate and showed little change up to about passage level 25 after which doubling times became progressively longer, consistent with *in vitro *senescence.

Cell counts were measured in duplicate in a hemocytometer every 24 h for 96 h. The counts were plotted on a log scale and were fitted to a straight line by linear regression. The standard error in the counts with respect to the line of regression was less than 2.5% for all curves. The correlation coefficients of all the growth lines were in the range 0.91–0.93.

## Abbreviations

GSH = reduced glutathione, GSSG = oxidized glutathione, NAC = N-acetylcysteine, pRb = retinoblastoma protein, ROS = reactive oxygen species

## Authors' contributions

All authors contributed equally to preparing the paper.

## Conflict of interest

Three of the authors (HF, LMS, & MB) hold stock in Redoxia Israel, Ltd, that may stand to gain from the publication of this manuscript, and Redoxia Israel has applied for patents related to its contents.
